# Introducing rapid diagnostic tests for malaria into drug shops in Uganda: design and implementation of a cluster randomized trial

**DOI:** 10.1186/1745-6215-15-303

**Published:** 2014-07-29

**Authors:** Anthony K Mbonye, Pascal Magnussen, Clare IR Chandler, Kristian S Hansen, Sham Lal, Bonnie Cundill, Caroline A Lynch, Siân E Clarke

**Affiliations:** School of Public Health, Makerere University and Ministry of Health, Box 7272, Plot 6 Lourdel Street Nakasero, Kampala, Uganda; London School of Hygiene and Tropical Medicine, Keppel Street, London, WC1E 7HT UK; Institute of International Health, Immunology and Microbiology, University of Copenhagen, Øster Farimagsgade 5, building 22-23, 1353 Copenhagen, K, Denmark

**Keywords:** Malaria, Rapid diagnostic tests, ACT, Drug shops, Private sector, Pragmatic trial, Uganda

## Abstract

**Background:**

An intervention was designed to introduce rapid diagnostics tests for malaria (mRDTs) into registered drug shops in Uganda to encourage rational and appropriate treatment of malaria with artemisinin-based combination therapy (ACT). We conducted participatory training of drug shop vendors and implemented supporting interventions to orientate local communities (patients) and the public sector (health facility staff and district officials) to the behavioral changes in diagnosis, treatment and referral being introduced in drug shops. The intervention was designed to be evaluated through a cluster randomized trial. In this paper, we present detailed design, implementation and evaluation experiences in order to help inform future studies of a complex nature.

**Methods:**

Three preparatory studies (formative, baseline and willingness-to-pay) were conducted to explore perceptions on diagnosis and treatment of malaria at drug shops, and affordable prices for mRDTs and ACTs in order to inform the design of the intervention and implementation modalities. The intervention required careful design with the intention to be acceptable, sustainable and effective. Critical components of intervention were: community sensitization and creating awareness, training of drug shop vendors to diagnose malaria with mRDTs, treat and refer customers to formal health facilities, giving pre-referral rectal artesunate and improved record-keeping. The primary outcome was the proportion of patients receiving appropriately-targeted treatment with ACT, evaluated against microscopy on a research blood slide.

**Results:**

Introducing mRDTs in drug shops may seem simple, but our experience of intervention design, conduct and evaluation showed this to be a complex process requiring multiple interventions and evaluation components drawing from a combination of epidemiological, social science and health economics methodologies. The trial was conducted in phases sequenced such that each benefited from the other.

**Conclusions:**

The main challenges in designing this trial were maintaining a balance between a robust intervention to support effective behaviour change and introducing practices that would be sustainable in a real-life situation in tropical Africa; as well as achieving a detailed evaluation without inadvertently influencing prescribing behaviour.

**Trial registration:**

NCT01194557 registered with ClinicalTrials.gov 2 September 2010.

## Background

A key malaria control strategy in Sub-Saharan Africa is to reduce malaria-related mortality and severe morbidity through early diagnosis and effective case management [1]. In up to 80% of malaria cases, care is provided outside the formal health sector and polypharmacy is common [2, 3]. Shops selling anti-malarial drugs are numerous, easily accessible, more oriented towards satisfying consumer needs, less prone to drug stock-outs than government health facilities and are therefore often visited as the first source of treatment [2–5]. Strategies to ensure prompt effective treatment with artemisinin-based combination therapy (ACT) in Africa need to take into account the role of drug shops in management of malaria and to ensure that appropriate anti-malarial drugs are sold to patients [6].

The World Health Organization (WHO) recommends that management of malaria should include confirmation, either microscopically or with an antigen-based rapid diagnostic test (mRDTs), of all suspected malaria cases before treatment with relatively costly ACT [6]. Rapid diagnostic tests to confirm malaria infection are reported to be accurate, simple to use with minimal training and potentially cost-effective in the context of ACT use [7–10]. However, experience has shown that while mRDTs are relatively simple technologies, their introduction as a tool for diagnosis is not straightforward [11–17]. With the majority of malaria cases being ‘self-diagnosed’ by the patient and treated with drugs purchased from a local shop, changes in diagnostic practice are not only needed amongst public healthcare providers, but also amongst patients themselves, pharmacies and drug shops. The availability of diagnostic confirmation in drug shops may increase willingness to pay for new ACT treatment since customers would clearly know whether they have malaria or not.

A study was conducted to examine the feasibility and effectiveness of introducing mRDTs into registered drug shops in Uganda to encourage rational and appropriate treatment of malaria. Study outcomes were anti-malarial drug prescription practices in drug shops, as well as acceptability and demand for improved diagnosis, purchase of mRDTs and use of ACTs. The primary trial endpoint was the proportion of patients receiving appropriately-targeted treatment with ACT, evaluated against microscopy on a research blood slide collected at the time of consultation. The study also aimed to examine the economic consequences of diagnostic testing for households, including the cost of initial consultation and medicines purchased at the drug shop, as well as any subsequent treatment-seeking behaviour within the following 14 days. It was hypothesized that the use of diagnostic testing would reduce over-diagnosis and over-prescription of anti-malarial drugs in the private sector. It was further hypothesized that the availability of diagnostic confirmation of malaria would increase uptake and sale of ACT (rather than monotherapy or cheaper, less efficacious, anti-malarial drug combinations), and would also encourage purchase of the full treatment dose, as well as patient adherence to the full course of ACT.

This paper reports the design, implementation and methods of evaluation of the intervention study and summarizes it under seven themes, namely: i) the study setting, ii) characteristics of the recipients, iii) the content of the intervention, iv) characteristics of those delivering the intervention, v) the mode of delivery, vi) the intensity and duration, and vii) methods to evaluate adherence to intervention (fidelity to treatment protocols) as previously recommended [18, 19]. Specific considerations when undertaking an intervention trial in the context of drug shops in Sub-Saharan Africa are discussed.

## Methods

### The study setting

The study was conducted in Mukono District in central Uganda. Like most of Uganda, the district is endemic for malaria and the majority of the population (88%) live in rural areas. The total population of the district is 850,900 with an annual growth rate of 2.3% and predominantly consists of subsistence farmers of the Baganda ethnic group. The policy in Uganda supports public-private partnerships to improve health outcomes and registered drug shops are thus supposed to participate in the prevention and treatment of malaria [20].

To inform the design of the intervention, a series of formative research studies were undertaken, including focus group discussions (with community members, health workers and drug shop vendors), a baseline survey of registered drug shops in the study area and exit interviews with customers. In addition, a willingness-to-pay survey was also conducted. The findings from this formative research are published in full elsewhere [21–24], but we highlight key findings pertinent to the design of the intervention below.

### Characteristics of the recipients (registered drug shops and drug shop customers)

In Uganda, registered drug shops are licensed to sell non-prescription drugs, including anti-malarial drugs but not antibiotics or injections. They are expected to be operated by a qualified health worker and fulfill minimum building standards and are subject to periodic inspection by the District Assistant Drug Inspector (DADI), with a license renewable annually for a fee. A total of 59 registered drug shops within 20 geographical clusters were eligible to participate in the trial and were invited to attend a training workshop, the characteristics of which were recorded in the baseline survey [21, 22]. Most were located in peri-urban areas and rural trading centers and were typically comprised of one to two rooms with one to two staff. On the day of the baseline survey, the majority of drug shop staff present were female, most had attained secondary level education, and approximately 50% were qualified health workers (state enrolled nurse or above). Nonetheless, few had previously received any specific training on the management of malaria or use of mRDTs, and knowledge on first-line treatment for malaria was low. All drug shops reported being open at least five days a week, with the majority (68%) open every day. Opening hours were long, with many shops open up to 10 pm at night, consistent with other findings that most trade occurs in the evening [25]. Typically, drug shop vendors (DSVs) reported seeing around 10 febrile patients each day (range 2 to 50 patients). The formative research undertaken with community members, drug shop vendors (DSVs) and health officials highlighted the need for the intervention design to respond to the character of registered drug shops, sometimes trusted and distrusted by the community, and their dual role as both folk practitioners and providers of modern biomedicine [23].

Since the intervention aimed at improving the diagnosis of malaria and prescription practices of DSVs, these were also recipients of the intervention. Their characteristics have been described above. For the intervention to be successful, patients had to be present at the drug shops for the diagnostic testing to be performed. The possibility that customers come to buy drugs for others was an initial concern. The exit interviews were helpful in confirming that in the majority of cases, the patient came to the drug shop in person to seek treatment for a fever, and that diagnostic testing was indeed feasible. Patients seeking treatment for fever at registered drug shops ranged in age from 1 month to 76 years, with approximately a third being children under the age of 5 years and a further third being adults above 18 years. These data were useful in understanding the treatment-seeking patterns and consultation rates at drug shops. This helped to design the patient follow-up procedure and to estimate the number of patients who were likely to receive an mRDT, ACT and/or pre-referral rectal artesunate. Microscopic examination of blood samples taken during the exit interview were used to determine the proportion of febrile patients with malaria parasites that purchased an ACT at baseline, as well as over-prescription to parasite-negative patients, and to inform sample size calculations for the primary endpoint of the trial (appropriate ACT treatment). The qualitative research revealed that mRDTs were not wholly unfamiliar to community members, and importantly that there was a pre-existing perception that not all fevers are malaria, and that diagnostic testing was viewed as potentially helpful in reducing this uncertainty [22, 23]. However, the qualitative findings showed that there were concerns in the community that mRDTs could test for HIV and that the purchasing advice given by the DSV might be influenced by a desire to get profit from drug sales. These findings were used to identify key messages for information leaflets that were used to raise awareness in the community of the purpose of malaria diagnostic testing and its availability in government facilities and registered drug shops.

### The content of the intervention

**Figure 1:**
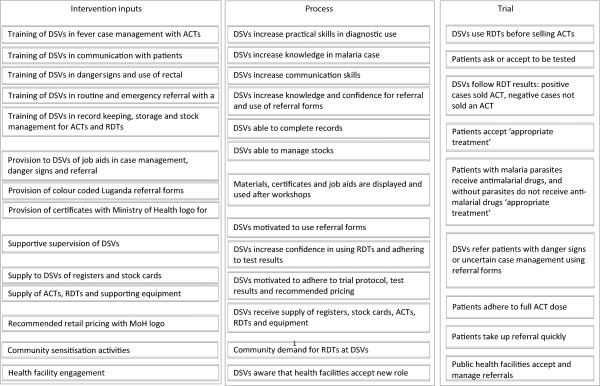
**Logical model to explain elements of the intervention.**

The content of the intervention was based on findings from the formative research of public health officials, non-governmental organization (NGO) staff and researchers and policymakers with previous experiences of implementing interventions with DSVs in Uganda. This helped to develop the underlying principles for the intervention (Table 1, Figure 1) and to situate the intervention in the context of other public health programs. The intervention was designed to address key deficiencies and challenges to appropriate diagnosis and treatment in drug shops, such as the lack of training in national malaria treatment guidelines, lack of reference materials (guidelines, job aids), limited record-keeping in shops and weak linkages with the public health system, which had been identified during the formative research. The intervention arm received training to cover the rationale for diagnostic testing in febrile patients, performing an mRDT and interpretation of the test result, while drug shop vendors (DSVs) in the control arm were trained in presumptive diagnosis of malaria.

**Table 1:** **Components and content of the intervention in the mRDT and control arms**

Intervention component	Intervention (mRDT) arm	Control arm
Training of DSVs	Four-day interactive training workshops covering practical skills, knowledge and communication skills in the following topics:	Three-day interactive training workshops covering practical skills, knowledge and communication skills in the following topics:
	• The new role of DSVs with ACT and mRDT	• The new role of DSVs with Coartem
	• How to receive clients, confirm fever and start recording	• How to receive clients, assess fever and start recording
	• Performing and reading an mRDT and blood slides	• How recognize and assess clients with signs of severe illness
	• How to recognize clients with signs of severe illness	• Taking a blood slide
	• How to treat clients who are mRDT-positive, including treatment of uncomplicated malaria with Coartem and treatment and referral of severe malaria including rectal artesunate suppositories	• How to treat clients with fever
	• How to deal with clients who are mRDT-negative, including referral and explaining negative mRDT results	• How to check for any other signs of illness
	• Keeping records, storage and monitoring mRDT and ACT	• Keeping records, storage and monitoring of Coartem
Job aids	Take-home materials: flow chart, job aid incorporating mRDTs, artesunate and referral forms; Danger r signs	Take-home training manual; flow chart job aid incorporating presumptive treatment, artesunate and referral; referral job aid for presumptive treatment; danger signs job aid for presumptive treatment
Referral forms	Supply of referral forms for sending with clients to health facilities, colour-coded for routine or emergency referral, incorporating mRDT results	Supply of referral forms for sending with clients to health facilities, colour-coded for routine or emergency referral
Certification	A4 certificate detailing completion of course in *‘*Use of Artemisinin-based combination therapies and Rapid diagnostics tests for home-based management of fever in Uganda’ with Ministry of Health logo	A4 certificate detailing completion of course in *‘*Use of Artemisinin-based combination therapies for home-based management of fever in Uganda’ with Ministry of Health logo
Supportive supervision	Initial 3-month period of intense support with regular weekly visits conducted by project staff with on-the-spot feedback on Coartem and mRDT use, record-keeping and other project- related topics	Initial 3-month period of intense support with regular weekly visits conducted by project staff with on-the-spot feedback on Coartem use, record-keeping and other project- related topics
Registers and stock cards	Supply of registers with treatment record forms for each client including mRDT recording; supply of stock cards to monitor supply of Coartem (yellow and blue), mRDTs, rectal artesunate and blood slides	Supply of registers with treatment record forms for each client; supply of stock cards to monitor supply of Coartem (yellow and blue), rectal artesunate and blood slides
Supplies	Distribution of mRDTs and Coartem by the study team either by direct delivery or visits from DSVs to the project office. Continuous supply was ensured through visits and mobile phone communication between DSVs and project staff.	Distribution of Coartem by the study team either by direct delivery or visits from DSVs to the project office. Continuous supply was ensured through visits and mobile phone communication between DSVs and project staff.
Recommended retail pricing	Pricing lists for mRDTs and Coartem provided to DSVs with Ministry of Health logo	Pricing lists for Coartem provided to DSVs with Ministry of Health logo
Advertising placard	Each DSV was given a placard to place in the roadside near their shop to advertise the availability of malaria diagnostics.	No placards were given.
Community sensitization	Meetings within 8 sub-counties (in which the 20 clusters were located) to meet political leaders and get their consent; training of sub-county trainers, identifying Village Health Teams (VHTs) who would carry out community sensitization; and to organize training of VHTs.	
SOPs	Distribution and training in standard operating procedures for project activities including completion of records, making blood slides, storage of project-related materials, logistics of supplies and collections for blood slides	
Supplies of project materials	Supply of materials for carrying out blood slides for the trial evaluation, including gloves, lancets, slides and storage, sharps boxes. Routine collection of blood slides and sharps boxes, ensuring supply of materials required.	
Health facility	Sensitization about project	
	Health units in the study area were visited and made aware of the referrals from DSVs and requested to keep records on the referrals.	

Legend:

mRDT Rapid Diagnostic Test for malaria.

DSV Drug Shop Vendor.

ACT Artemisinin-based Combination Therapy.

VHT Village Health Teams.

Coartem Artemisinin/lumefantrine.

#### Development of a training manual, job aids and standard operating procedures

The trainer’s manual and accompanying job aids were designed by the research team to highlight the importance of appropriate treatment of malaria with ACT as the first line anti-malarial drug in accordance with the Ministry of Health (MOH) malaria management guidelines. Additional job aids were developed by the team, including for a treatment algorithm, and summary guidance on detecting danger signs and referring patients. Training and job aids focused on recognizing the signs of severe malaria and administration of rectal artesunate as a pre-referral treatment, followed by referral to a health facility for specialized care. It was also recognized that in some circumstances there might be need to refer patients who had other symptoms and/or were mRDT-negative to a health facility, and guidelines were provided on when to consider referral. However, in recognition that many DSVs were qualified health workers with varied levels of training and experience, these guidelines were not intended to be prescriptive, allowing drug shop vendors with the knowledge and means to provide other non-malaria treatments. The manual also covered issues relevant to patient safety, including safe disposal of sharps and bio-waste, storage conditions and expiration dates. In addition to the job aids, drug shop vendors were provided with treatment registers, referral forms, and stock cards to support good practice in stock management. Simple record forms were designed so as to not be too time consuming to complete, and to respect the privacy of DSV business transactions. All forms were translated into the local language, which included the referral form, with the hope that this would help patients understand why they were being referred, and was considered a facilitatory factor for acceptability and uptake of referral advice by patients.

The training manual was based on a curriculum that enabled theory and practice, encouraging interactive discussion and reflective practice, supported by practical exercises and role-plays. The pictorial job aids and recording forms were distributed and used by DSVs during these exercises. Role plays were devised to practice the communication skills necessary to obtain a relevant clinical history, to explain the outcome of the mRDT test, reason for treatment given, and where appropriate, referral advice. Two versions of the manual and report forms were produced for each arm of the trial, which differed only in the method of malaria diagnosis to be used (control treatment based on signs and symptoms only or mRDT-based). In all other respects, the content of the intervention and the training were identical in order that the attribution of effect could be based on the use of mRDTs rather than the accompanying training, materials and activities.

### The intensity of the intervention (supporting interventions)

The intervention also aimed at addressing challenges that drug shops were expected to face in implementing diagnostic testing, for example recognizing that DSVs would need to broker this change with communities. Thus in addition to the technical skills to improve diagnosis and treatment, DSVs were also trained in negotiation and communication skills. The design of the training manual was based on adult learning principles, intending to be interactive and participatory. Supporting interventions were also undertaken to enhance community acceptability of mRDTs through the provision of leaflets to village health teams summarizing key messages and community sensitization to change. At the end of the training, DSVs were presented with a certificate with the MOH logo in the presence of the District Health Team and local community leaders to confirm their new status as a trained malaria treatment provider. Drug shops in the mRDT arm were also given a placard to place at the roadside advertising the availability of malaria diagnostic tests at their premises. The training, certification and community sensitization activities were also intended to legitimize the role of registered drug shops as approved providers of malaria diagnosis and treatment, and so minimize distrust and skepticism towards DSVs by the community and other health providers.

### The mode of delivery of the intervention

#### Community sensitization

Prior to implementation, a meeting was held with the District Health Team (DHT) to share objectives and the scope of the study. This was followed by meetings within eight sub-counties (in which the 20 clusters were located) to meet political leaders and obtain their agreement for the trial to be undertaken in their locality. Community sensitization was undertaken through a process of cascade training using existing district health structures. Sub-county trainers were orientated on the aims and objectives of implementing RDT in registered drug shops and procedures to be introduced. The specific roles of the trainers were to identify Village Health Teams (VHTs) who would carry out community sensitization, organize a one-day training of VHTs and supervise the subsequent community sensitization. The VHTs were trained before the start of the trial to inform communities within the study area about the study, its purpose and what to expect. The information given to participants during community sensitization activities was summarized in the information leaflet for VHTs and translated into the local language (Luganda). Both the sub-county trainers and the research team supervised VHTs on a weekly basis. Health units in the study area were visited and made aware of the intervention in drug shops, the possibility of referrals from DSVs and requested to keep records on patients referred.

#### Randomization and DSV training

There is an increased risk of randomization failure in cluster randomized trials with a small number of clusters, with an increased possibility that study arms are unbalanced with respect to potential confounders, undermining the credibility of trial findings. To counter this risk, and to balance cluster-related factors that might *a priori* be associated with the primary outcome or expected to influence the effect of the intervention between the two study arms, restricted randomization informed by the baseline survey was used to allocate eligible drug shop clusters to the intervention. A total of 20 clusters were randomized to the control or the intervention arm (10 per arm). All registered drug shops located in eligible clusters were invited to attend a training workshop in malaria diagnosis and treatment, and informed written consent was obtained from drug shop owners who attended the training, agreeing to participate in the trial and its evaluation. Training workshops for drug shop vendors (DSVs) in the intervention arm lasted four days, to include topics relating to mRDTs specifically. In the control arm, DSVs were invited to a three-day workshop with the same content and interactive style but without training on mRDTs. The training was modular (see Table 1 for topics) and was conducted by a team composed of the District Health Educator (DHE), the Principal Investigator from the Ministry of Health and the Field Coordinator.

### Adherence to the study protocols (supervision)

After commissioning the intervention, for an initial period of two months immediately after training DSVs were provided with additional close support supervision (in both arms) to assist with the adoption of the new diagnosis and treatment procedures into their everyday practice with intervention arm, and record- keeping and quality of blood slides in the control arm. This comprised weekly site visits conducted by project staff to support DSVs to implement the intervention and also to encourage accurate and complete record-keeping. A supervisory checklist was used and issues and questions raised were recorded (Table 2. The DSVs were given on-the-spot feedback and a date for next supervision agreed on. Intense support supervision ended at the end of December 2010, with a reduction in the number and frequency of site visits (Table 3). In this way, we sought to provide the additional support required to achieve behaviour change whilst recognizing the financial and logistic constraints to supervision under realistic programmatic conditions with limited funding.

**Table 2:** **Some issues raised by drug shop vendors during supervision**

Some issues raised by drug shop vendors during supervision	
•	What will the drug shop vendors do when the study ends?
•	Due to poverty, there are some people who still want to buy less tablets of ACT and they end up taking an under-dose
•	Some caretakers leave patients at home and come to buy anti-malarial drugs, hence it can be difficult to get blood for testing
•	Some patients fear to be pricked for the blood sample
•	Some patients/caretakers asked for the results of the research slide (control arm)
•	Some patients with danger signs do not comply when referred because they do not receive adequate care at health units
•	The referral forms are ignored and despised by the staff at health facilities because they are written in Luganda (local language)
•	After hearing about mRDT some people also came for other tests like HIV, typhoid and syphilis.

**Table 3:** **Supply management of mRDT and drugs**

Supply management of mRDT and drugs	
•	Procure mRDT and drugs
•	Quality assurance of drugs and mRDT (arrange lot testing and design and implement system for field testing of mRDT)
•	Detailed plan for supply management (how requests are made, frequency and who supplies)
•	Monitoring and action on leakage of drugs and mRDT

#### Pricing and stock management

The trial was designed to uphold the principle enshrined in the Affordable Medicines Facility for Malaria (AMFm) that to assure access to effective malaria treatment, ACTs need to be affordable for the majority of the population. During the trial, DSVs were therefore provided the mRDTs and ACT treatments free of charge, and expected to sell these at a low cost to their customers. Recommended retail prices for mRDTs and ACT were informed by the level of proposed AMFm subsidy and site-specific willingness-to-pay study [24]. The recommended retail prices for mRDTs and ACT were agreed with DSVs at the time of training, but were not advertised to the community, as we wished to monitor price adherence. However, later on, drug shops did request that they be given an official recommended retail price list to show to customers at time of purchase.

#### Logistics and stock management

DSVs were supplied with an initial stock of mRDTs and ACT, but thereafter were expected to collect their supplies from a project office established at Mukono town, as required. This was considered realistic since DSVs would come to town periodically to replenish other drug stocks. The project office was staffed with a dedicated project officer to provide support and supervision to the DSVs, keep stock of drugs, mRDTs and other supplies, and coordinate data management of completed treatment forms and research blood slides on a daily basis, as well as a microscopist and two data clerks. During the formative research, baseline and final evaluation surveys, four to five experienced social scientists were recruited and trained to conduct the focus group discussions, client exit interviews and household interviews. A vehicle and driver were provided to enable drug shop visits during the phase of close support supervision and later evaluation surveys. Key logistical issues in supply management are listed in Table 3.

### Evaluating the impact of the intervention

#### Evaluation of the outcomes

Evaluation of the intervention to assess the primary and secondary endpoints was undertaken towards the end of the first year of implementation (Table 4). This allowed practices to normalize before evaluation and to prevent research activities influencing provider behaviour (Table 5). The primary outcome of the intervention was the proportion of patients receiving appropriately-targeted treatment with ACT, defined as an ACT provided to malaria blood side parasite-positive patients. In the mRDT arm, blood was taken from consenting patients to perform both an mRDT and a thick blood film. In the control arm only a thick blood film was taken. The blood films were transported to the Vector Control Division of the Ministry of Health in Kampala and examined by a laboratory technician. The small number of febrile patients typically seen each day meant that patient exit interviews during the baseline survey proved costly and time consuming. In addition, the majority of customers are often seen in the evening, introducing practical constraints and risks of sampling bias. These constraints, coupled with the concern that exit interviews might also influence provider behaviour through increasing compliance with the treatment guidelines during the period of observation (Hawthorne effect [25]), influenced our choice of evaluation methods during the trial. Likewise, we did not attempt to directly observe consultations. We therefore evaluated provider behaviour (adherence to test result, treatment given, referral) primarily through the establishment of a routine reporting system. DSVs were provided with carbon-copy treatment and referral registers: one copy was retained by the DSV, one submitted to the project office, and in the case of referrals, one copy was also given to the client. We felt it was realistic to expect private providers participating in a program with subsidized commodities to contribute data to a health management information system (HMIS), and that routine reporting could also support accountability and stock management. The impact of the intervention was thus evaluated on the basis of the mRDT result and anti-malarial treatment recorded in patient treatment records routinely completed by DSVs on patients seen during a full 12-month period after the end of the close support supervision. This was in order to capture the full range in seasonal variation in consultation and mRDT positivity rates, under conditions of routine implementation (with very limited supervision and external influence). Nonetheless, the evaluation of treatments given was based on self-reported data, and could be vulnerable to selective and/or inaccurate reporting by DSVs.

**Table 4:** **The study timelines**

Study activity	Time period
Formative research	May - July 2010
Baseline survey	June - August 2010
Randomization	September 2010
Training	October - November 2010
Intense supervision	November - December 2010
Evaluation phase	January - December 2011
Household surveys	September 2011 - June 2012

**Table 5:** **Outcomes, sources of data and type of analyses**

Endpoint	Source of data	Planned analyses
**Primary endpoints:** Appropriate treatment	Treatment record form	The proportion of patients receiving appropriate treatment will be calculated as follows: (number of patients who are slide-positive and were given a 1^st^-line anti-malarial + the number of patients who are slide-negative and not given a 1^st^-line anti-malarial) divided by total febrile consultations.
Over-prescription	Treatment record form Accessibility to ACT and mRDTs	Proportion of patients who are not parasite-positive (slide-negative), who receive inappropriate ACT treatment from a drug shop, with the proportion of incorrectly treated malaria cases being based on the ‘gold standard’ of a research blood slide.
Provider adherence with mRDT result	Treatment record form	Proportion of patients who receive appropriate ACT treatment, consistent with mRDT result. Depending on the severity of symptoms an mRDT-positive patient is expected to have received either artemether-lumefantrine (Coartem) tablets or a rectal artesunate suppository.
Cost-effectiveness	Treatment record form, Implementation costs,	Incremental societal cost per additional case of appropriate treatment resulting from introducing mRDT in drug shops:
	Day 14 household visits	Incremental costs of the mRDT intervention will be calculated by subtracting the societal costs in the current practice arm from the societal costs in the mRDT arm. The incremental effect will be measured as the difference in the number of cases receiving appropriate treatment (primary endpoint) in the current practice arm and the mRDT arm.
**Secondary endpoints:** Prompt appropriate treatment	Treatment record form	Proportion of patients seen at a registered drug shop who receive appropriate ACT treatment within 24 hours of onset of malaria symptoms
Referral: appropriate management of mRDT-negative patients	Treatment record form	Appropriate treatment of mRDT-negative patients (lack of anti-malarial sale and provision of referral.
Referral: timeliness and uptake of referral by patients	Referral forms, referral follow-up visits	Timeliness and uptake of referral at a health unit by referred patients. The mean time interval between referral and uptake for patients taking up the severe referral will be compared between arms
Patient adherence to ACT	Day 4 patient follow-up visit,	Proportion of patients followed up on day 4 that were prescribed ACT and took the full 3 day dose in the correct manner. Adherence to treatment is based on examination of the blister pack, if available, and the patient/caregivers report of how the treatment was taken during day 4 patient follow-up interview.
Equity of diagnosis	Day 14 household visits	Proportion of patients with fever receiving a diagnostic test for malaria, compared across socio-economic groups. Denominator: Households with at least one person with a history of fever within the last 14 days.
Equity of treatment	Day 14 household visits	Proportion of patients with malaria (slide-positive), receiving treatment with an ACT, compared across socio-economic groups. Denominator: Households with at least one person with a history of fever within the last 14 days.
Acceptability of mRDT to patients	Treatment record form (refusals), Day 4 patient follow-up visit, Focus group discussions	Proportion of patients with fever accepting to purchase a diagnostic test; proportion of ACT sales preceded by a positive test.
Perceptions of ACT, mRDTs and acceptability of the intervention (community, drug shops health staff)	Focus group discussions	Data from focus group discussions and key informant interviews will be transcribed and transferred to NVivo version 8 (QSR International); a software program for the management and analysis of qualitative data. Coding of the transcripts will take place through an iterative process. Initially data will be grouped into themes drawn from idea codes to generate a ‘node tree of ideas’.

Legend:

mRDT Rapid Diagnostic Test for malaria.

DSV Drug Shop Vendor.

ACT Artemisinin-based Combination Therapy.

NVivo qualitative data analysis software; QSR International Pty Ltd. Version 8, 2008.

In order to assess this, a number of other methods were used to provide corroborative data to assess the validity of the reports, including the collection and re-reading of mRDTs to assess the inter-rater reliability of interpretation and recording of mRDT results by DSVs. Studies in the private sector often use simulated client visits, in which mystery shoppers perform specific tasks such as measuring quality of service, compliance with regulation or gathering specific information about products and services [26]. The use of stimulated client visits was not feasible as a means of evaluating the main outcome of our study, since this required a sample of blood to be collected from the client. Nevertheless, a small number of simulated client visits were conducted to assess adherence to treatment guidelines in specific situations where a patient might decline a blood test or the patient is not present for testing.

Towards the end of the first year of implementation, interviews were also conducted with a random sample of 500 patients at their place of residence 10 to 14 days after the initial drug shop consultation. In addition to providing an independent confirmation of the treatment received, price adherence by DSVs and elicitation of patient views on the acceptability of the diagnosis and services at drug shops, this follow-up also aimed to capture the full costs of treatment borne by the household (through inclusion of costs incurred both at the time of initial consultation and further treatment-seeking undertaken in the subsequent two weeks, no later than 14 days after the initial consultation minimize recall bias). Patient follow-up interviews also provided data on patient adherence to the full three-day course of ACT treatment and active surveillance for adverse events. To obtain a random sample, field assistants approached DSVs according to a randomized schedule and obtained a list of all patients who were treated with ACT in the previous four days and thus eligible for follow-up. Tracing patients proved laborious, with interviewees in paid employment and in peri-urban areas more likely to be absent at the time of the home visit. To mitigate risk of loss to follow-up and potential bias, repeat visits were attempted and telephone contacts and addresses of households were recorded routinely by the DSVs during the evaluation phase and used to make appointments for follow-up by research assistants.

### Evaluating the cost-effectiveness of the intervention

A cost-effectiveness component was designed with the aim of comparing the cost per correctly treated fever patient in the intervention and the control arm. Data on costs as well as effectiveness measured as correctly treated patients (using microscopy as gold standard) by study arm were captured. Unit costs of malaria treatment in public health centers were also captured in order to incorporate patients seeking additional care in public health centers after visiting a drug shop. Recurrent and capital costs borne by the health service for sensitizing the community, training DSVs, supervising DSVs, purchasing and distributing ACT and mRDTs and other supplies required for delivering the intervention were estimated. Evaluation of the cost-effectiveness component was undertaken from a societal perspective and costs included: a) those related to deployment of the intervention and borne by the health service, and b) those incurred by the household when seeking treatment for fever, including the direct costs of transport, diagnosis and drugs and opportunity costs of lost time and production.

### Ethics

Ethical approval for the trial was granted from review boards at the Uganda National Council of Science and Technology (reference: HS 546) and the London School of Hygiene and Tropical Medicine. At the time of eliciting informed consent to participate in the trial, DSVs were informed that mystery drug shop visits might take place. Written informed consent was from drug shops sought prior to all interviews.

## Results and discussion

### Coordination of a multi-disciplinary research team

The main challenge in designing this trial was its complex nature that necessitated a combination of epidemiological, clinical, social science and health economics methodologies. We assembled a team of researchers with diverse skills as this trial required strong team work, good collaboration and harmonized decision-making.

### Balancing robust interventions with real-life situations

Another challenge was maintaining a balance between a robust intervention and introducing practices that would be sustainable in a real-life situation for example, introducing records for DSVs aimed at evaluating the intervention. However, we were mindful of the work load put on staff and privacy attached to the customers, price levels and the variety of drugs stocked. Similarly, the supervision of DSVs was done with care not to be misunderstood as inspection and control. To maintain a constant supply of mRDTs and ACT at drug shops, DSVs would visit the project office to bring treatment forms and pick up supplies whenever they came to town to do other errands. This provided a periodic opportunity for DSVs to raise any challenges and resolve these individually with the project supervisor on an ongoing basis. Mobile phones were also used for communication with the project supervisor for replenishing stocks and to solve field problems.

### Sustainability and future scale-up of the intervention

The intervention was designed with a view to keeping implementation and supervision costs to a minimum, conforming to approaches typically used by the Ministry of Health, such as utilizing networks of community health volunteers for community sensitization and cascade training. Close support supervision was limited to an initial period of two months. To address potential issues of sustainability during the scale-up of the intervention, we involved the DHT members and community leaders from the inception of the study, to secure local political support and ownership amongst influential stakeholders. The DHE and the district nursing officer participated in the early visits to the study areas to meet with community leaders and select sub-county trainers. The DHE was also involved in designing training materials, pre-testing them and the training of DSVs. Similarly, the DADI participated in the verification of registered DSVs and reviewing training materials, especially the referral forms.

### Monitoring for contamination

In this trial we could not restrict patient choice in where they chose to seek treatment, and the implications of treatment-seeking by patients needed to be considered when interpreting study findings. To minimize mixing (crossover) of patient populations, a cluster randomized design was used, such that drug shops in close geographical proximity were randomized to the same arm. Nevertheless, since factors other than geographical proximity may influence which private provider a patient chooses to frequent, we cannot assume that all patients resident in the same parish visited the same cluster of drug shops. Polypharmacy is the norm in many parts of Sub-Saharan Africa, and it is also possible that during the course of treatment-seeking one patient may visit more than one source of treatment. Typically, almost half of patients had first sought treatment elsewhere, and this could have included seeking treatment from registered drug shops in clusters in a different arm, or from other drug outlets not participating in the trial. Therefore it was important to collect data on the village and parish of residence to examine geographical patterns in treatment-seeking and to monitor for crossover. By examining where patients chose to go for treatment, we were also able to directly monitor the acceptability of the intervention to patients. Data on prior and subsequent treatment-seeking was collected in a sub-sample of customers from both arms to allow monitoring of this issue. Data was also collected on whether any staff at the drug shop worked elsewhere, and if so, where and how often.

### Dropout of DSVs

Since high costs were noted to be a constraint to patients in the study area and likely to affect appropriate treatment of febrile and non-febrile illnesses, meetings were held with DSVs to discuss affordable prices for ACT and mRDTs. This discussion was guided by a study on willingness-to-pay for an mRDT and a course of ACT [24]. Four drug shops in the intervention arm and two in the control arm dropped out before training because they thought the intervention would not work and therefore did not attend the training. This led to one cluster in the mRDTs arm to drop out and the sample size was recalculated. Later in 2011, two DSVs in the intervention arm dropped out. One shop closed down and the other changed ownership and the new worker did not wish to participate in the study. In the control arm seven dropped out due to closure of business and shifting to other locations. Drug shops differed in the size of their client base, reflecting differences in location and popularity. This was included as a balance criterion in the restricted randomization, but differing levels of patient recruitment (cluster size) also needed to be considered in analysis. The typically small number of staff meant that some shops closed down temporarily during periods of sickness or maternity leave, reducing sample size in some clusters.

### Monitoring provider behaviour

A key outcome of the study was to monitor whether DSVs would adhere to the mRDT results and provide appropriate treatment according to the guidelines. Concerns had been voiced during the formative research about the potential for profit motive to affect case management, especially adherence to the treatment guidelines to not sell an ACT in the face of negative mRDT results. None of the evaluation methods were ideal, and all carried some risk that DSVs might modify their behaviour in the knowledge that adherence to guidelines was being monitored. To minimize this risk, we used routine self-reported data, with additional validation data collected using mystery shoppers and follow-up interviews with a sub-sample of patients timed to occur only after the first full 12-monthsof implementation was completed. Another concern was whether DSVs would be motivated to refer customers into the public health system, particularly given our formative research findings of their difficult relationship with the formal system. To address these issues a qualitative sub-study was designed to document the treatment and referral of customers by DSVs to formal health facilities through follow-up interviews with customers and focus group discussions with DSVs, local health facility staff and patients who had been referred.

### Local initiatives by drug shops

In working with the commercial sector, the possibility that some private providers might use the intervention to promote their business which may inadvertently affect the delivery of the intervention should not be overlooked. For example, we found that in addition to the initial community sensitization undertaken as part of the planned intervention, some DSVs independently utilized local radios to advertise the intervention, or advertised their services in community meetings, church and women’s groups. In some cases, DSVs also chose to advertise the price of mRDTs and ACTs. This was considered an innovation and we subsequently attempted to document this for each DSV in order to assess any possible influence this had on the success of patient recruitment, as part of the process evaluation.

### Context of the intervention

The context in which a trial occurs needs also be considered. For example, treatment-seeking for malaria treatment and acceptability of mRDT testing at drug shops are both likely to be influenced by diagnostic and treatment practices in local health facilities, as well as by temporal fluctuations in the availability of mRDTs and ACTs in the public sector. From the baseline study, DSVs were not stocking and using mRDTs [22]. To stabilize this effect on study endpoints over the duration of trial, we found it was necessary to periodically supplement government supplies of mRDTs and ACTs to avoid stock-outs, which had major cost implications for the study. To understand the broader social context, we also undertook a discourse analysis to examine how drug shops were usually portrayed in the national media and policy documents over the time period of the study.

Within the trial itself, as part of the evaluation of this complex intervention a number of contextual aspects were investigated to situate the intervention within the overall public health goal of increasing access to prompt effective malaria treatment. In the study, epidemiological, economic and socio-behavioral analyses were undertaken to provide a comprehensive evaluation. The results of these studies will help policymakers to make a decision on the deployment of ACT and mRDTs in home-based management of malaria in different settings. Research findings will also help to identify how to achieve maximal coverage of correct and appropriate malarial treatment and to minimize misuse of ACT.

## Conclusions

This trial aimed to answer a ‘simple’ policy-relevant question of whether mRDT should be introduced at drug shops. The design of the intervention and the trial to evaluate this was far from simple, and our account of this here illustrates the network of people, materials, resources, protocols and logistics that need to be coordinated for such an intervention trial to be enacted.

## Abbreviations

ACT: Artemisinin-combination therapy
AMFm: Affordable medicines facility for malaria
DADI: District assistant drug inspector
DHE: District health educator
DHT: District health team
DSV: Drug shop vendor
HMIS: Health management information system
mRDT: Rapid diagnostic tests for malaria
MOH: Ministry of health
WHO: World health organization
VHT: Village health team.
